# RASSF6 Expression in Adipocytes Is Down-Regulated by Interaction with Macrophages

**DOI:** 10.1371/journal.pone.0061931

**Published:** 2013-04-23

**Authors:** Yohei Sanada, Takahiro Kumoto, Haruna Suehiro, Fusanori Nishimura, Norihisa Kato, Yutaka Hata, Alexander Sorisky, Noriyuki Yanaka

**Affiliations:** 1 Department of Molecular and Applied Bioscience, Graduate School of Biosphere Science, Hiroshima University, Higashi-Hiroshima, Japan; 2 Department of Dental Science for Health Promotion, Hiroshima University Graduate School of Biomedical Sciences, Hiroshima, Japan; 3 Department of Medical Biochemistry, Graduate School of Medicine, Tokyo Medical and Dental University, Tokyo, Japan; 4 Chronic Disease Program, Ottawa Hospital Research Institute, Departments of Medicine and of Biochemistry, Microbiology and Immunology, University of Ottawa, Ottawa, Ontario, Canada; University of Bari, Italy

## Abstract

Macrophage infiltration into adipose tissue is associated with obesity and the crosstalk between adipocytes and infiltrated macrophages has been investigated as an important pathological phenomenon during adipose tissue inflammation. Here, we sought to identify adipocyte mRNAs that are regulated by interaction with infiltrated macrophages *in vivo*. An anti-inflammatory vitamin, vitamin B6, suppressed macrophage infiltration into white adipose tissue and altered mRNA expression. We identified >3500 genes whose expression is significantly altered during the development of obesity in db/db mice, and compared them to the adipose tissue mRNA expression profile of mice supplemented with vitamin B6. We identified *PTX3* and *MMP3* as candidate genes regulated by macrophage infiltration. PTX3 and MMP3 mRNA expression in 3T3-L1 adipocytes was up-regulated by activated RAW264.7 cells and these mRNA levels were positively correlated with macrophage number in adipose tissue *in vivo*. Next, we screened adipose genes down-regulated by the interaction with macrophages, and isolated *RASSF6* (*Ras association domain family 6*). RASSF6 mRNA in adipocytes was decreased by culture medium conditioned by activated RAW264.7 cells, and RASSF6 mRNA level was negatively correlated with macrophage number in adipose tissue, suggesting that adipocyte RASSF6 mRNA expression is down-regulated by infiltrated macrophages *in vivo*. Finally, this study also showed that decreased RASSF6 expression up-regulates mRNA expression of several genes, such as *CD44* and *high mobility group protein HMGA2*. These data provide novel insights into the biological significance of interactions between adipocytes and macrophages in adipose tissue during the development of obesity.

## Introduction

The metabolic syndrome is linked to visceral obesity and is a major risk factor for insulin resistance, type2 diabetes, and cardiovascular disease [1], [2]. Adipose tissues respond to nutrient excess through increases in adipocyte size (hypertrophy) and cell number (hyperplasia). Adipocyte hypertrophy and hyperplasia can both lead to adipose tissue expansion and a variety of effects, including hypoxia, adipocyte cell death and enhanced secretion of a large number of bioactive substances, adipocytokines [3], [4]. Obese adipose tissue is characterized by dynamic alterations in cellular composition and function, and chronic low-grade inflammation [3]–[7]. Dramatic changes in stromal cell number and cell type in adipose tissue point to a pathological role for immune cells as a contributor to chronic inflammation, including macrophages, T lymphocytes and mast cells [3]–[7]. In particular, recent reports emphasize macrophage accumulation in expanding adipose tissue as a key phenomenon [5], [8] and raise important questions about the molecular mechanisms underlying macrophage infiltration and the influence of adipose tissue macrophages on energy homeostasis and inflammatory responses related to obesity-induced insulin resistance [1], [2], [9].

It is increasingly clear that macrophage infiltration into adipose tissue is essential for adipose tissue inflammation. Mice lacking monocyte chemoattractant protein-1 (Mcp1/Ccl2) or its receptor, CC motif chemokine receptor-2 (CCR-2), have decreased macrophage accumulation in adipose tissue and inflammation in fat and attenuated insulin resistance during a high fat diet [10], [11]. Additionally, conditional ablation of macrophages using the CD11c promoter resulted in a significant reduction of macrophage accumulation in the adipose tissue of obese mice on a high fat diet, accompanied by the normalization of insulin sensitivity [12]. These observations suggest that inflammatory signals from adipose tissue macrophages play a critical role in chronic inflammation, including proinflammatory activation of adipocytes and/or the disruption of adipocyte functions, and this has drawn attention to potential interactions between adipocytes and macrophages in obese adipose tissue. Recent work using an *in vitro* coculture system demonstrated the possibility that adipocytes and macrophages communicated via production of a large number of biological substances, including fatty acids, tumor necrosis factor-α (TNF-α), growth factors and reactive oxygen species [13]–[16]. Although these *in vitro* observations strongly suggest that crosstalk between adipocytes and macrophages in obese adipose tissue is crucial for chronic inflammation and adipose tissue remodeling, the potential for crosstalk *in vivo* remains unclear.

Vitamin B6 is a water-soluble vitamin essential for normal growth, development and metabolism [17], [18]. Dietary vitamin B6 has been shown to have anti-inflammatory effects, including the prevention of contact dermatitis and stomatitis, and recent studies also suggest that vitamin B6 is an effective nutritional therapy for chronic inflammatory diseases [19]–[21]. In this study, we examined the effect of dietary vitamin B6 on chronic inflammation in the adipose tissue of mice fed a high fat diet, and show that vitamin B6 supplementation suppressed macrophage infiltration into adipose tissue, accompanied by a decrease of adipose mRNA expression including macrophage markers, without alteration of other immune cells, such as CD8^+^ T cells and mast cells. We analyzed mRNA expression in adipose tissue of the leptin receptor-deficient obese mouse model using DNA microarray analysis, and confirmed that numerous genes related to macrophage infiltration are significantly up-regulated. We considered that characterization of these two transcriptomes would be highly informative for investigating the molecular basis of the *in vivo* crosstalk between adipocytes and macrophages, and would lead to the discovery of novel adipose tissue genes tightly associated with macrophage infiltration. We were particularly interested in adipocyte functions affected by macrophages based on a DNA microarray analysis of genes expressed differentially in 3T3-L1 adipocytes co-cultured with RAW264.7 macrophages *in vitro.* We further screened adipocyte genes that respond to treatment with activated macrophages amongst candidate genes based on *in vivo* observations. This led us to identify RASSF6 (Ras association domain family 6) and we showed that its mRNA expression in adipocytes was decreased in obese mice and in the presence of activated macrophages *in vitro*, suggesting that cellular functions of RASSF6 in adipocytes are regulated through macrophage interactions. Therefore, our comparison strategy is useful to identify adipocyte-derived molecules that are closely related to macrophage infiltration in obese adipose tissue and to improve the understanding of the molecular basis of crosstalk between adipocytes and adipose tissue macrophages *in vivo*.

## Materials and Methods

### Animals and Diets

The animal study was approved by the Hiroshima University Animal Committee (Permit Number: C10-3), and the mice were maintained in accordance with the Hiroshima University guidelines for the care and use of laboratory animals. All surgery was performed under ether anesthesia, and all efforts were made to minimize suffering. Male CD-1 (ICR): Crj mice (4 weeks old, Charles River Japan, Hino, Japan) were housed in groups of 2 or 3 in metal cages in a room with controlled temperature (24±1°C) and a 12 h light/dark cycle; light from 0800 to 2000, daily. They had free access to stock diet and deionized water. After consuming a commercial stock diet (MF, Oriental Yeast, Tokyo, Japan) for 1 week, the total of 24 mice were divided into 2 groups of 12 mice. The basal diet was composed of the following components (g/kg diet): α-cornstarch, 302; casein, 200; sucrose, 200; corn oil, 200; cellulose, 50; AIN-93G mineral mixture, 35; AIN-93 vitamin mixture (vitamin B6 free), 10; and L-cystine, 3. Vitamin B6 (pyridoxine (PN) hydrochloride, Nacalai Tesque, Kyoto, Japan) was supplemented to the basal diet at concentrations of either 1 mg/kg or 35 mg/kg for 8 weeks. Food intake and body weight were measured daily. Male db/db (BKS.Cg-m^+/+^ Lepr db/J, 7 weeks old) were obtained from Charles River. db/db mice (n = 3) and db/+ mice (n = 3) were subjected to a DNA microarray analysis.

### Cell Culture

Mouse 3T3-L1 preadipocytes and mouse macrophage RAW264.7 cells were cultured in a maintenance medium (10% fetal bovine serum, 100 units/ml penicillin and 100 µg/ml streptomycin in Dulbecco’s modified medium (DMEM)) at 37° in 5% CO_2_/95% humidified air. Confluent 3T3-L1 cells were treated with differentiation medium (maintenance medium plus 0.5 mM 3-isobutyl-1-methylxanthine (IBMX), 5 µg/ml insulin, and 1 µM dexamethasone (DEX), MDI) and incubated for 2 days. Then, differentiation medium was replaced with growth medium (maintenance medium supplemented 5 µg/ml insulin), which was refreshed every 2 days. 3T3-L1 cells and RAW264.7 cells were co-cultured in maintenance medium using a transwell system (Corning Inc., Acton, MA, USA) with a 0.4-µm porous membrane. 1×10^5^ differentiated 3T3-L1 cells were cultured in the lower chamber, whereas 5×10^4^ RAW264.7 cells were cultured in the upper chamber, and thereafter, stimulated with 1 µg/ml of LPS for 24 hr. Stealth siRNA duplex oligoribonucleotides against mouse RASSF6 were synthesized by Invitrogen. The sequences were as follows: sense 5′-AAUGUAAAGAGCGAAAUCCCGAGGG-3′, antisense 5′-CCCUCGGGAUUUCGCUCUUUACAUU-3′. 3T3-L1 cells were transfected with these siRNAs to a final concentration of 20 nM using LipofectAMINE RNAimax (Invitrogen).

### Isolation of Stromal Vascular Fraction Cells and Adipocytes

Epididymal white adipose tissue isolated from male HFD mice was minced in phosphate-buffered saline and digested with 1 mg/ml collagenase Type I (Worthington Chemical Corporation) for 30 min at 37°C. The resulting cell suspension was filtered through a 100-µm filter and centrifuged at 233×*g* for 1 min to separate adipocytes from stromal vascular fraction (SVF) cells.

### DNA Microarray

Total RNAs were isolated from epididymal white adipose tissue using RNeasy lipid tissue kit (Qiagen Sciences, Germantown, MD), and pooled RNAs were subjected to cRNA synthesis for a DNA microarray analysis according to the manufacturer’s instructions (44K whole mouse genome 60-mer oligo microarray, Agilent Technologies, Palo Alto, CA). All procedures of fluorescence labeling, hybridization, slide, and image processing were carried out according to the manufacturer’s instructions. In this experiment, each comparison was hybridized to two arrays employing a DyeSwap method in order to eliminate the bias between dyes because the difference between Cyanine 3-CTP (Cy-3) and Cyanine 5-CTP (Cy-5) altered the efficiency of hybridization in the case of the competitive DyeCoupling assay. Gene expression data were obtained and statistically analyzed using Agilent Feature Extraction software, using defaults for all parameters except ratio terms, which were changed according to the Agilent protocol to fit the direct labeling procedure. Files and images, including error values and *P* values, were exported from the Agilent Feature Extraction Program (version 9.5). The microarray data were deposited in the NCBI GEO data base (available on the World Wide Web at www.ncbi.nlm.nih.gov/geo) under accession number GSE43465.

### RT-PCR Analyses

Semi-quantitative and quantitative PCR analyses were performed on total RNAs prepared with an RNeasy lipid tissue kit. The reverse transcriptase reaction was carried out with 1 µg total RNA as a template to synthesize cDNA using ReverTra Ace (TOYOBO, Osaka, Japan) and random hexamers (TOYOBO), according to the manufacturer’s instructions. For semi-quantitative PCR analysis, cDNA and primers were added to the GoTaq Master Mix (Promega, Madison, WI, USA) to give a total reaction volume of 20 µl. The reactions were sampled after 30 cycles under different PCR conditions, to monitor product accumulation. For quantitative PCR analysis, cDNA and primers were added to the THUNDERBIRD SYBR qPCR Mix (TOYOBO), to give a total reaction volume of 15 µl. PCR reactions were then performed using StepOnePlus™ (Applied Biosystems, Foster City, CA). Conditions were set to the following parameters: 10 min at 95°C, followed by 45 cycles each of 15 s at 95°C and 1 min at 60, 62, or 64°C. The primers used for PCR analyses were as follows: TNF-α, forward, 5′-CCGATGGGTTGTACCTTGTC-3′, and reverse, 5′-CGGACTCCGCAAAGTCTAAG-3′; MCP-1/CCL2, forward, 5′-GGTCCCTGTCATGCTTCTGG-3′, and reverse, 5′-CCTTCTTGGGGTCAGCACAG-3′; Spp1, forward, 5′-ATTTGCTTTTGCCTGTTTGG-3′, and reverse, 5′-CTCCATCGTCATCATCATCG-3′; Msr1, forward, 5′-TCAAACTCAAAAGCCGACCT-3′, and reverse, 5′-ACGTGCGCTTGTTCTTCTTT-3′; Emr1, forward, 5′-ATTGTGGAAGCATCCGAGAC-3′, and reverse, 5′-GTAGGAATCCCGCAATGATG-3′; PTX3, forward, 5′-TGGGTGGAAAGGAGAACAAG-3′, and reverse, 5′-CCGATCCCAGATATTGAAGC-3′; MMP3, forward, 5′-TGGAGATGCTCACTTTGACG-3′, and reverse, 5′-AGAGCTGCACATTGGTGATG-3′; Rassf6, forward, 5′-CCATAAGCAGGGAACAACTC-3′, and reverse, 5′-AGGTTTCCGGTGTGTTCAAC-3′; Hmga2, forward, 5′-AGCAAGAGCCAACCTGTGAG-3′, and reverse, 5′-CGAGGATGTCTCTTCAGTCTCC-3′; CD44, forward, 5′-CCGAGGATTCATCCCAACGC-3′, and reverse, 5′-GCCGCTGCTGACATCGTCAT-3′; β-actin, forward, 5′-TTGGGTATGGAATCCTGTGGCATC-3′, and reverse, 5′-CGGACTCATCGTACTCCTGCTTGC-3′; Adiponectin, forward, 5′-ACAGGAGATGTTGGAATGACAG-3′, and reverse, 5′-CTGCATAGAGTCCATTGTGGTC-3′.

### Immunohistochemical Analysis

The epididymal adipose tissue was isolated and fixed with neutral buffered formalin and embedded in paraffin. An immunohistochemical study was carried out using 4-µm-thick paraffin-embedded sections for the macrophage marker F4/80. The number of F4/80-positive cells in more than 100 serial fields was counted in a blinded fashion through the microscope, and the data were obtained as the mean number/mm^2^.

*Statistical analyses-* Values are presented as means ± S.E. Statistical significance was determined by one-way ANOVA and Duncan’s multiple-range test. Differences were considered significant for *p*<0.05 (*) and *p*<0.01 (**).

## Results

### Effects of Dietary Vitamin B6 on Adipose Tissue Gene Expression

**Figure 1:**
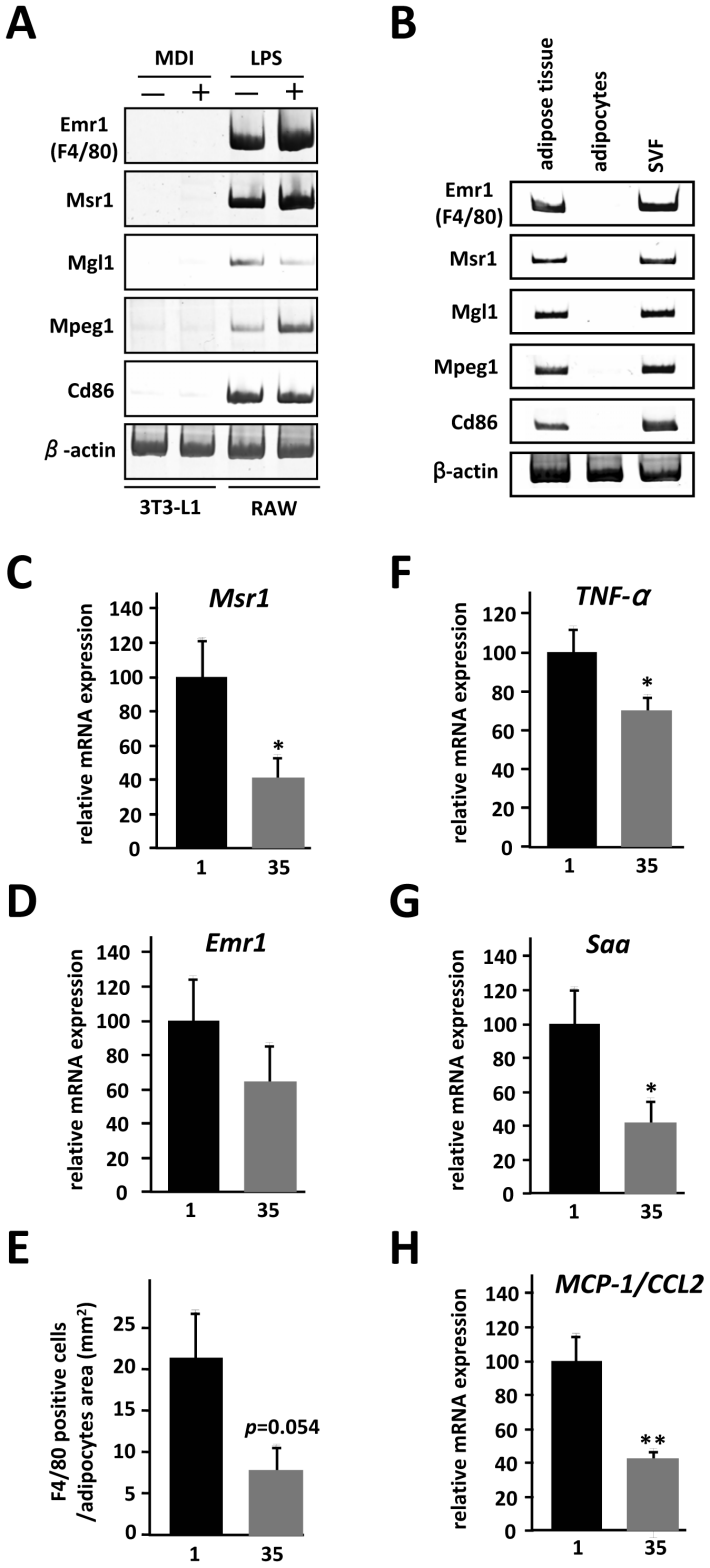
Effects of dietary vitamin B6 on adipose gene expression in HFD mice. *A*,*B*, Semiquantitative RT-PCR was performed to determine mRNA levels of genes related to macrophages. 3T3-L1 preadipocytes were treated with MDI for 48 h and differentiated into mature adipocytes as described under “Materials and Methods”. RAW264.7 cells were stimulated with 1 µg/ml of LPS for 18 hr. Mature adipocytes and SVF were isolated from white adipose tissue of HFD mice as described under “Materials and Methods”. The level of Emr1 (F4/80) transcript was used as a control for macrophages. *C*,*D*,*F–H*, Total RNA from individual mice (n = 12) in two groups was subjected to quantitative PCR to examine mRNA expression level of selected genes. All values are normalized to β-actin levels. The data (mean ± S.E.) are representative of three independent experiments. *P<0.05, **P<0.01 compared with those of 1 mg/kg PN (*1*). *E*, The number of F4/80-positive cells in the epididymal adipose tissue was examined by F4/80 immunostaining as described under “Materials and Methods”.

Vitamin B6 supplementation has been demonstrated to have an anti-inflammatory effect by both animal and epidemiological studies [19]–[21]. The first objective of this study was to explore the relationship between vitamin B6 activity and chronic inflammation of white adipose tissue in mice fed a high-fat diet. We examined the levels of vitamin B6 in epididymal white adipose tissues of CD-1 mice in response to dietary supplementation. As shown in Table S1, we showed that 35 mg PN HCl/kg diet increased the concentration of vitamin B6 (pyridoxal 5′-phosphate) by ∼5-fold in adipose tissues. Twenty-four mice were divided into two groups (n = 12) and fed either a 1 mg pyridoxine (PN) HCl/kg diet or a 35 mg PN HCl/kg diet for 8 weeks, since mRNA expression of macrophage marker genes was up-regulated in white adipose tissue in mice on the high-fat diet after 8 weeks (Fig. S1). Body weight, food intake and epididymal adipose tissue weight were not significantly different between the two groups (Table S2). We isolated total RNA from epididymal white adipose tissue of each group and compared gene expression profiles by DNA microarray data analysis. The data analysis indicated that 465 transcript levels were significantly down-regulated in the adipose tissue in response to vitamin B6 supplementation (*p*<0.05). In particular, we noticed that dietary vitamin B6 decreased mRNA expression of *EGF-like module-containing mucin-like hormone receptor-like 1* (*Emr1*), *macrophage scavenger receptor 1* (*Msr1*), *macrophage galactose N-acetyl-galactosamine specific lectin 1* (*Mgl1*), *macrophage expressed gene 1* (*Mpeg1*) and *Cd86*, which are considered markers of infiltrated macrophages (Table 1). In fact, as shown in Fig. 1A and 1B, we observed that these mRNAs are expressed in mouse macrophage RAW264.7 cells and SVF from epididymal white adipose tissue of HFD mice. To confirm the differential expression of these genes, total RNA from individual mice in the two groups were subjected to quantitative PCR. Supplemental vitamin B6 significantly decreased Msr1 mRNA expression in adipose tissue compared with the 1 mg PN HCl/kg diet group (Fig. 1C). In addition, vitamin B6 supplementation had a tendency to decrease *Emr1* mRNA expression (Fig. 1D, *p = *0.057) and the number of *F4/80*-positive cells (Fig. 1E, *p = *0.054) in adipose tissue. This study also showed that vitamin B6 supplementation significantly decreased tumor necrosis factor-α (TNFα), serum amyloid protein (SAA) and MCP-1/CCL2 mRNA levels in adipose tissue compared with the 1 mg PN HCl/kg diet group (Fig. 1F–H). The mRNA expression of individual mice was assessed using Pearson's correlation coefficient and showed a striking correlation between TNFα and SAA mRNA levels and number of *F4/80* positive cells (*r* = 0.619 and *r* = 0.497; *p*<0.01), respectively. It was noted that dietary vitamin B6 did not affect mRNA levels that are closely associated with mast cells and T lymphocytes, such as mast cell-specific proteases, Mcpts, Cd8a, CD4 and granzymes (data not shown). Recent findings indicate that there are two different polarization states of macrophages in obese adipose tissue [5], [22]; M1 or proinflammatory macrophages and M2 or anti-inflammatory macrophages. Our DNA microarray analysis demonstrated that vitamin B6 supplementation did not affect mRNA expression of arginase, IL-10 and Chi3l3 (chitinase 3-like 3), markers of M2 macrophages, but did down-regulate expression of CD11c, TNFα and MCP-1/CCL2, markers of M1 macrophages. However, vitamin B6 supplementation decreased Mgl1 mRNA level, a marker of M2 macrophages, suggesting that further experiments are needed to clarify the adipose macrophage polarization in the adipose tissue of mice with dietary vitamin B6. It was also noted that dietary vitamin B6 did not affect the adipocyte size of epididymal white adipose tissue (Fig. S2) or systemic glucose tolerance (Fig. S3). These observations, taken together, strongly suggest that the suppressive role of dietary vitamin B6 is specific to macrophage infiltration in the adipose tissue, and not mast cells nor T lymphocytes. We proceeded to use this intriguing feature of vitamin B6 supplementation to identify adipose-derived genes that are related to increased macrophage accumulation in obese adipose tissue *in vivo*.

**Table 1:** Effect of vitamin B6 supplementation on adipose tissue gene expression in mice fed a high fat diet.

Gene ID	Gene symbol	Gene description	Fold	P value
cytokines and chemokines				
NM_009987	Cx3cr1	chemokine (C-X3-C) receptor 1	0.46	0.000
NM_013652	Ccl4	chemokine (C-C motif) ligand 4	0.49	0.000
NM_009263	Spp1	secreted phosphoprotein 1	0.49	0.000
NM_011315	Saa3	serum amyloid A 3	0.49	0.000
NM_009915	Ccr2	chemokine (C-C motif) receptor 2	0.49	0.000
NM_009914	Ccr3	chemokine (C-C motif) receptor 3	0.51	0.000
NM_009917	Ccr5	chemokine (C-C motif) receptor 5	0.53	0.000
NM_013654	Ccl7	Mus musculus chemokine (C-C motif) ligand 7 (Ccl7),mRNA [NM_013654]	0.55	0.000
NM_021704	Cxcl12	chemokine (C-X-C motif) ligand 12	0.55	0.000
NM_013693	Tnf	tumor necrosis factor	0.55	0.000
NM_011333	MCP-1/Ccl2	chemokine (C-C motif) ligand 2	0.56	0.000
NM_008366	Il2	interleukin 2	0.58	0.002
NM_021274	Cxcl10	chemokine (C-X-C motif) ligand 10	0.58	0.000
NM_013653	RANTES/Ccl5	chemokine (C-C motif) receptor 5	0.59	0.000
NM_019418	Tnfsf14	tumor necrosis factor (ligand) superfamily, member 14	0.62	0.001
NM_011888	Ccl19	chemokine (C-C motif) ligand 19	0.67	0.001
NM_011337	Ccl3	chemokine (C-C motif) ligand 3	0.73	0.002
recruited monocyte/macrophage				
NM_145976	Trifab	TRAF-interacting protein with forkhead-associated domain,family member B	0.42	0.000
NM_010208	Fgr	feline sarcoma viral (Fgr) oncogene homolog	0.57	0.000
NM_178792	Sirpb1	signal-regulatory protein beta 1	0.58	0.000
NM_010387	H2-DMb1	histocompatibility 2, class II, locus Mb1	0.61	0.001
NM_019467	Aif1	allograft inflammatory factor 1	0.63	0.002
NM_010330	Emb	embigin	0.68	0.007
NM_139138	Emr4	EGF-like module hormone receptor-like sequence 4	0.71	0.021
cell adhesion and MMPs				
NM_010809	Mmp3	matrix metallopeptidase 3	0.32	0.000
NM_008605	Mmp12	matrix metallopeptidase 12	0.54	0.000
NM_021334	Itgax	integrin alpha X	0.57	0.000
NM_010576	Itga4	integrin alpha 4	0.58	0.000
NM_008607	Mmp13	matrix metallopeptidase 13	0.63	0.003
NM_008404	Itgb2	integrin beta 2	0.67	0.004
NM_008319	Icam5	intercellular adhesion molecule 5 (telencephalin)	0.68	0.007
NM_008401	Itgam	integrin alpha M	0.74	0.010
monocyte/macrophage markers				
NM_010821	Mpeg1	macrophage expressed gene 1	0.51	0.000
NM_031195	Msr1	macrophage scavenger receptor 1	0.56	0.000
NM_009853	Cd68	CD68 antigen	0.59	0.000
NM_010796	Mgl1	macrophage galactose N-acetyl-galactosamine specific lectin 1	0.62	0.000
NM_019388	CD86	CD86 antigen	0.67	0.005
NM_010130	Emr1	EGF-like module containing, mucin-like, hormone receptor 1	0.7	0.003
NM_030707	Msr2	macrophage scavenger receptor 2	0.71	0.018
inflammatory proteins				
NM_008987	Ptx3	pentraxin related gene	0.45	0.000

List of differentially expressed genes grouped into functional categories. DNA microarray analysis was repeated with the Cy3 and Cy5 dyes reversed (a dye swap), and fold change (*Fold*) represents the average of mRNA expression level in mice with a 35 mg PN HCl/kg diet relative to a 1 mg PN HCl/kg diet.

### Comparison of Genes from Two Transcriptomes Whose Expression is Regulated by the Interaction with Macrophages

**Figure 2:**
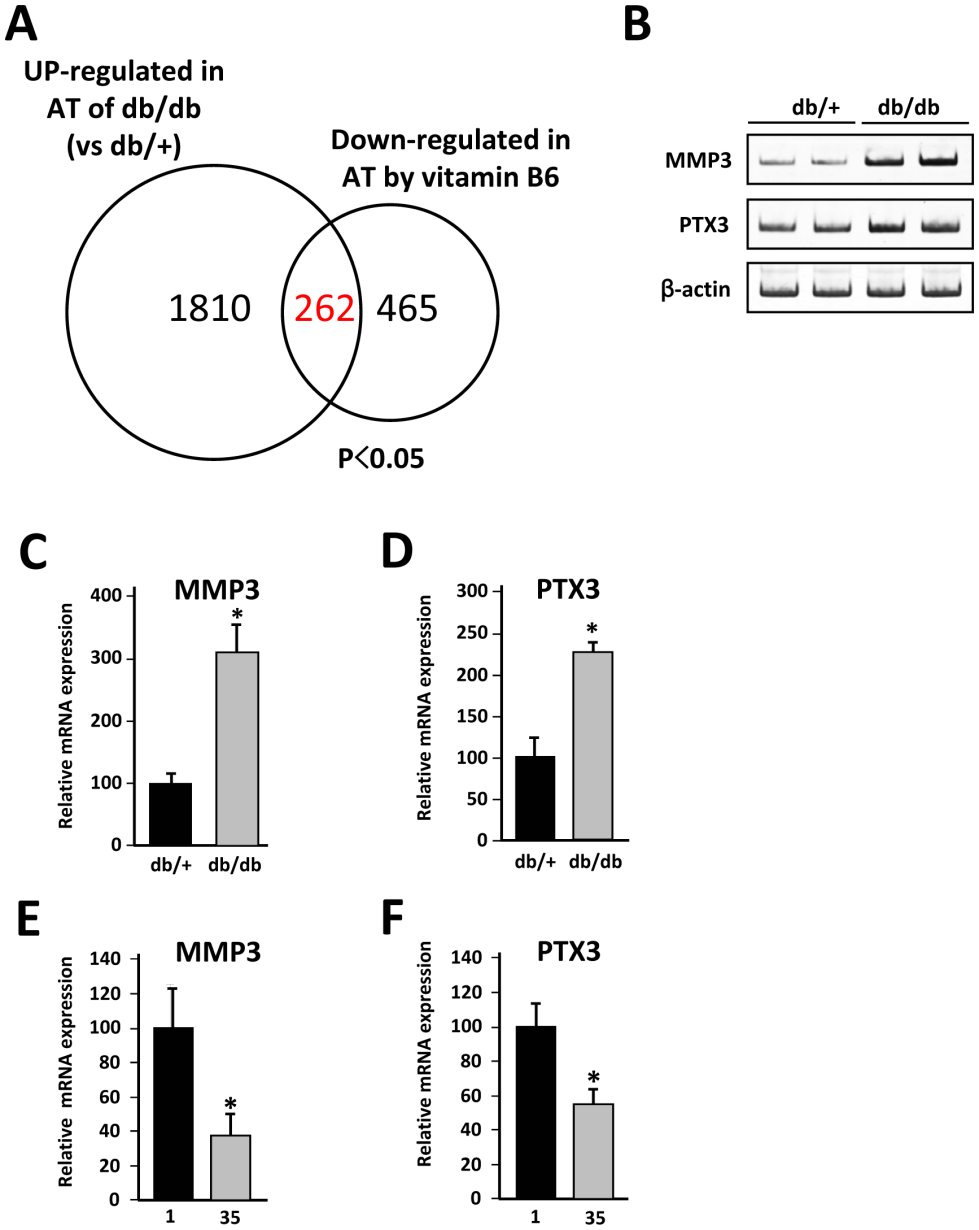
Analysis of two transcriptomes to isolate genes whose expression is upregulated by the interaction with macrophages. *A*, The Venn diagram shows genes that are upregulated in the db/db white adipose tissue and down-regulated by vitamin B6 supplementation. Of a total 1810 genes up-regulated in db/db adipose tissue, the expression of 262 genes was decreased by dietary vitamin B6 (*p*<0.05). *B*, Semiquantitative RT-PCR was performed to determine mRNA levels of *MMP3* and *PTX3*. The level of β-actin (*β-actin*) transcript was used as a control. *C*, *D*, Total RNA from individual mice (n = 4) in two groups was subjected to quantitative PCR to examine mRNA expression. All values are normalized to β-actin levels. The data (mean ± S.E.) are representative of two independent experiments. *P<0.05 compared with those of db/+ mice. *E*,*F*, Total RNA from individual mice (n = 12) in two groups was subjected to quantitative PCR to examine mRNA expression level. All values are normalized to β-actin levels. The data (mean ± S.E.) are representative of two independent experiments. *P<0.05 compared with those of 1 mg/kg PN (*1*).

**Figure 3:**
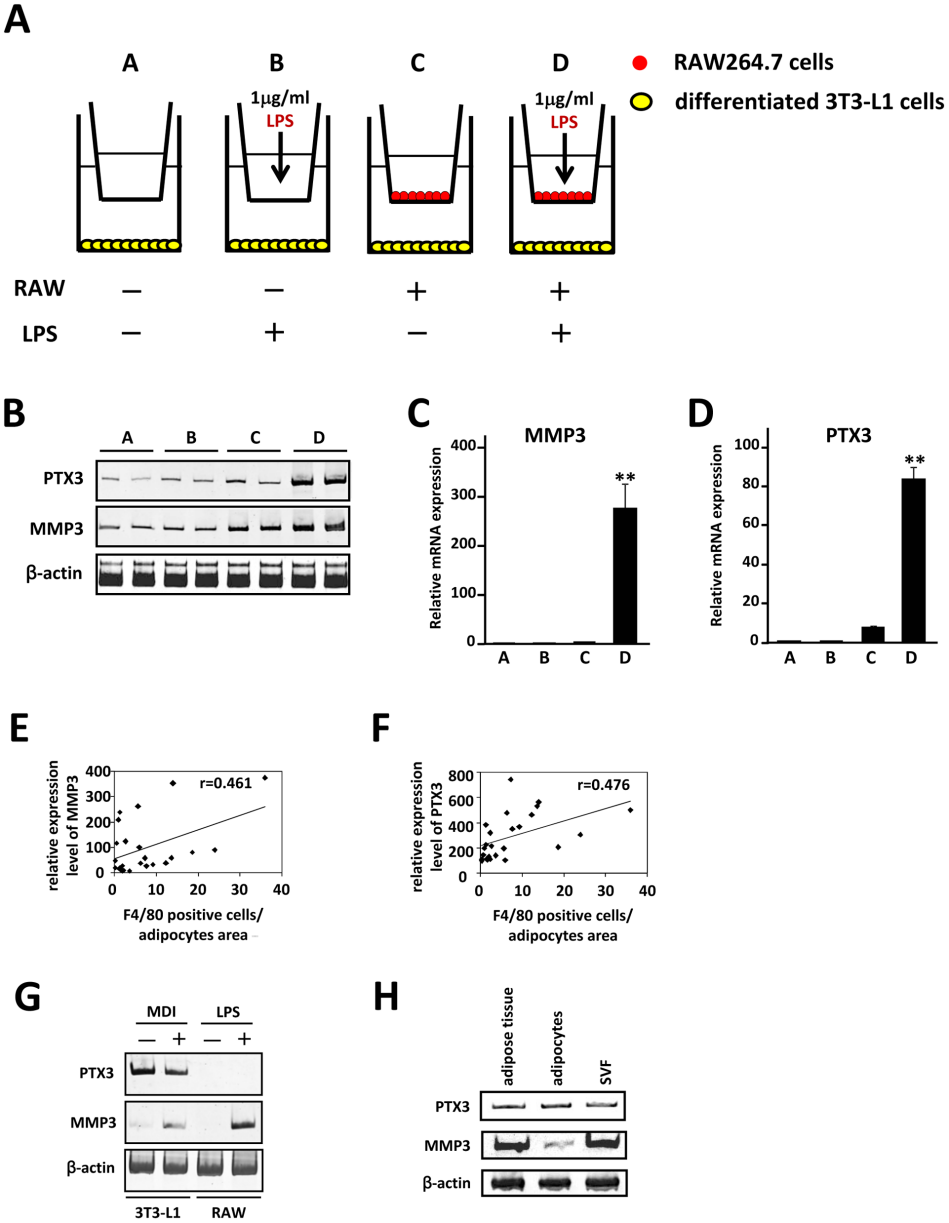
PTX3 and MMP3 expression in adipocytes is affected in the presence of activated macrophages. *A*, Illustration of the coculture system composed 3T3-L1 adipocytes and RAW264.7 cells is shown. *B*, Semiquantitative RT-PCR was performed to determine mRNA levels of *PTX3* and *MMP3*. The level of β-actin (*β-actin*) transcript was used as a control. *C*,*D*, Total RNAs from 4 groups was extracted and subjected to quantitative PCR to examine mRNA expression level of *PTX3* and *MMP3*. All values are normalized to β-actin levels. The data (mean ± S.E.) are representative of two independent experiments. **P<0.01 compared with those of group C. *E*,*F*, The relative mRNA expression level of each gene was determined by quantitative PCR and normalized to β-actin level. Pearson's correlation coefficient showed a positive correlation between MMP3 and PTX3 mRNA levels and number of *F4/80* positive cells in adipose tissue of mice fed HFD. *G*,*H*, Semiquantitative RT-PCR was performed to determine mRNA levels of PTX3 and MMP3. 3T3-L1 preadipocytes were treated with MDI for 48 h and differentiated into mature adipocytes as described under “Materials and Methods”. RAW264.7 cells were stimulated with 1 µg/ml of LPS for 18 hr. Mature adipocytes and SVF were isolated from white adipose tissue of HFD mice as described under “Materials and Methods”.

We hypothesized that characterization of the adipose gene expression profile with vitamin B6 supplementation would be highly informative for understanding the molecular basis of macrophage infiltration. As some research groups have previously reported [23], [24], we showed here that numerous gene clusters are altered in the db/db white adipose tissue. Since db/db mice and CD-1 mice (used in vitamin B6 experiments) are genetically different, we expected that the genes whose expression is up-regulated in the db/db white adipose tissue, but down-regulated by vitamin B6 supplementation, are common factors essential for macrophage infiltration into adipose tissue between two genetic backgrounds. Of a total 1810 genes up-regulated in db/db adipose tissue samples *(p*<0.05), the expression of 262 genes was decreased by dietary vitamin B6 (*p*<0.05, Fig. 2A). As might be predicted, within this group of 262 genes, macrophage marker genes such as *Emr1*, *Msr1* and *CD8*6 and several chemokines and chemokine receptor genes are included (Table 2). We further characterized adipocyte genes whose expression responds to macrophage infiltration into adipose tissue *in vivo*. Using our previous data [25] of mRNA expression profile of 3T3-L1 adipocytes when co-cultured with macrophage RAW264.7 cells *in vitro* for comparison, we selected adipocyte genes among the 262 candidate genes. We identified several candidate genes, including pentraxin 3 (PTX3) and matrix metalloproteinase-3 (MMP3). PTX3 and MMP3 mRNA expression was markedly increased in the db/db adipose tissue, whereas it was decreased in response to vitamin B6 supplementation (Fig. 2B–F). We proceeded to co-culture 3T3-L1 adipocytes with RAW264.7 cells (Fig. 3A) and showed that the PTX3 and MMP3 mRNA levels were markedly increased in the presence of macrophages activated by LPS treatment (Fig. 3B–D). Most importantly, a striking correlation was observed between MMP3 and PTX3 mRNA levels and number of *F4/80* positive cells in adipose tissue of HFD obese mice (Fig. 3E,F), strongly supporting the concept that adipose PTX3 and MMP3 mRNA levels are increased by the macrophage infiltration in adipose tissue *in vivo*. As shown in Fig. 3G,H, expression of PTX3 mRNA is highly expressed in 3T3-L1 cells (preadipocytes, adipocytes) and mature adipocytes fraction from white adipose tissue of HFD mice, whereas MMP3 is expressed in 3T3-L1 adipocytes, LPS-activated RAW264.7 cells and SVF, suggesting that increased MMP3 expression in obese adipose tissue may be derived from infiltrated macrophages.

**Table 2:** Analysis of two transcriptomes to isolate genes whose expression is upregulated by the interaction with macrophages.

Gene name	Fold 1 (db/db/db/+)	Fold 2 (35 mg/1 mg B6)
Spp1	547.85	0.49
Ccl2	4.27	0.60
msr1	6.64	0.56
Mgl1	8.36	0.62
Mpeg1	5.74	0.51
MMP3	6.08	0.32
PTX3	3.31	0.45

DNA microarray analysis was repeated with the Cy3 and Cy5 dyes reversed (a dye swap). Fold change (*Fold 1*) represents the average of mRNA expression level in db/db mice relative to db/+ mice. Fold change (*Fold 2*) represents the average of mRNA expression level in mice with a 35 mg PN HCl/kg diet relative to a 1 mg PN HCl/kg diet.

### Analysis of Two Transcriptomes Identifies Adipocyte Genes Whose Expression is Down-regulated by the Interaction with Macrophages

**Figure 4:**
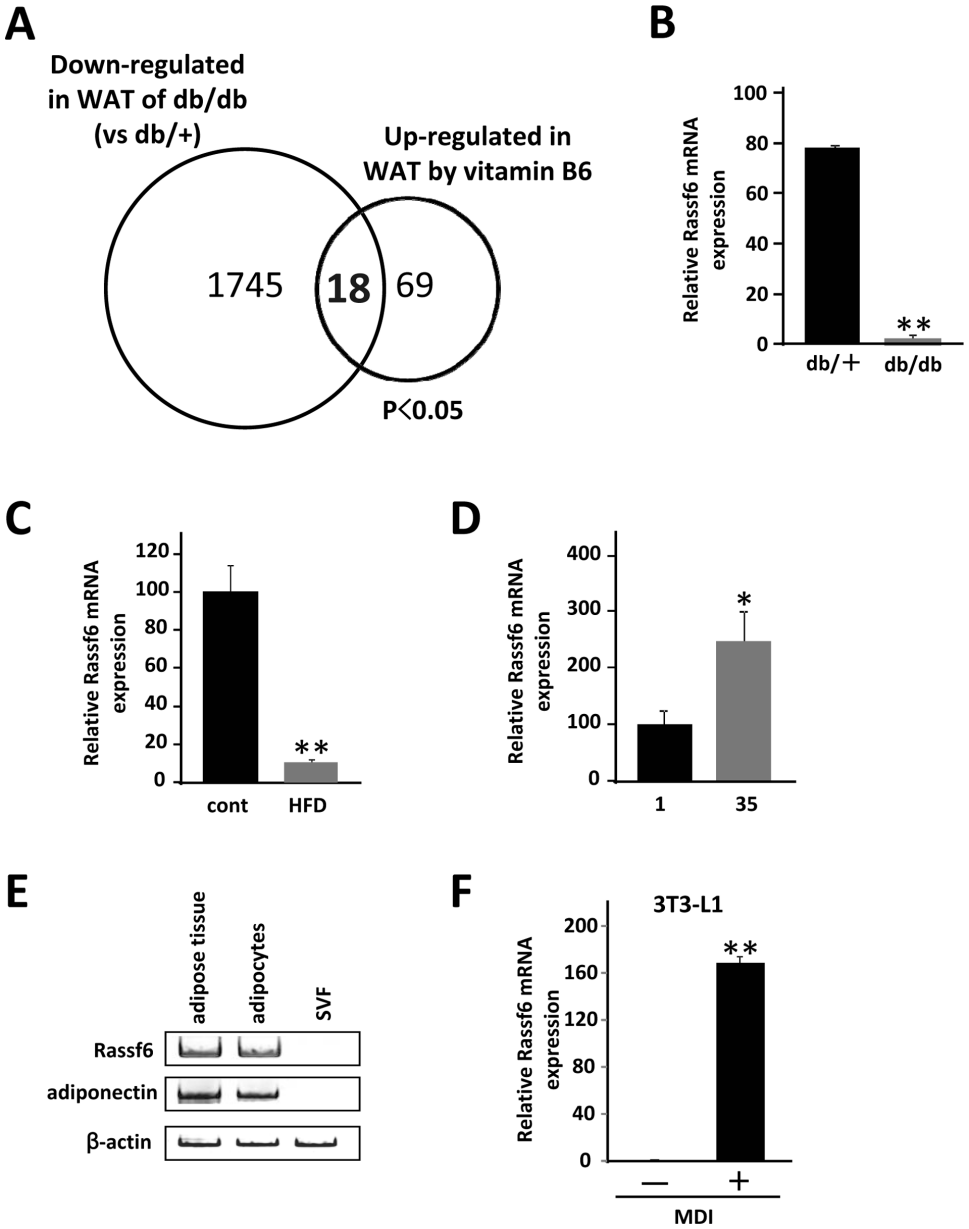
Analysis of two transcriptomes to isolate genes whose expression is downregulated by the interaction with macrophages. *A*, The Venn diagram shows genes that are downregulated in the db/db white adipose tissue and upregulated by vitamin B6 supplementation. Of a total 1745 genes downregulated in db/db adipose tissue, the expression of 18 genes was increased by dietary vitamin B6 (*p*<0.05). *B*, Total RNAs from individual mice (n = 3) were subjected to quantitative PCR. Values are normalized to β-actin levels. **P<0.01 compared with that of control mice (*db/+*). *C*, Mice were divided into two groups (n = 4), and fed basal diet (*cont*) or high fat diet (*HFD*) for 8 weeks (n = 4). The relative mRNA expression level of each gene was determined by quantitative PCR and normalized to β-actin level. **P<0.01 compared with that of mice with basal diet (*cont*). *D*, Total RNA from individual mice (n = 12) in two groups was subjected to quantitative PCR to examine RASSF6 mRNA expression level. All values are normalized to β-actin levels. *P<0.05 compared with that of 1 mg/kg PN (*1*). *E*, Mature adipocytes and SVF were isolated from white adipose tissue of HFD mice as described under “Materials and Methods”. The level of adiponectin transcript was used as a control for mature adipocytes. *F*, 3T3-L1 preadipocytes were treated with MDI for 48 h and differentiated into mature adipocytes as described under “Experimental Procedures”. Total RNA was extracted and subjected to quantitative PCR to examine RASSF6 mRNA expression level.

**Figure 5:**
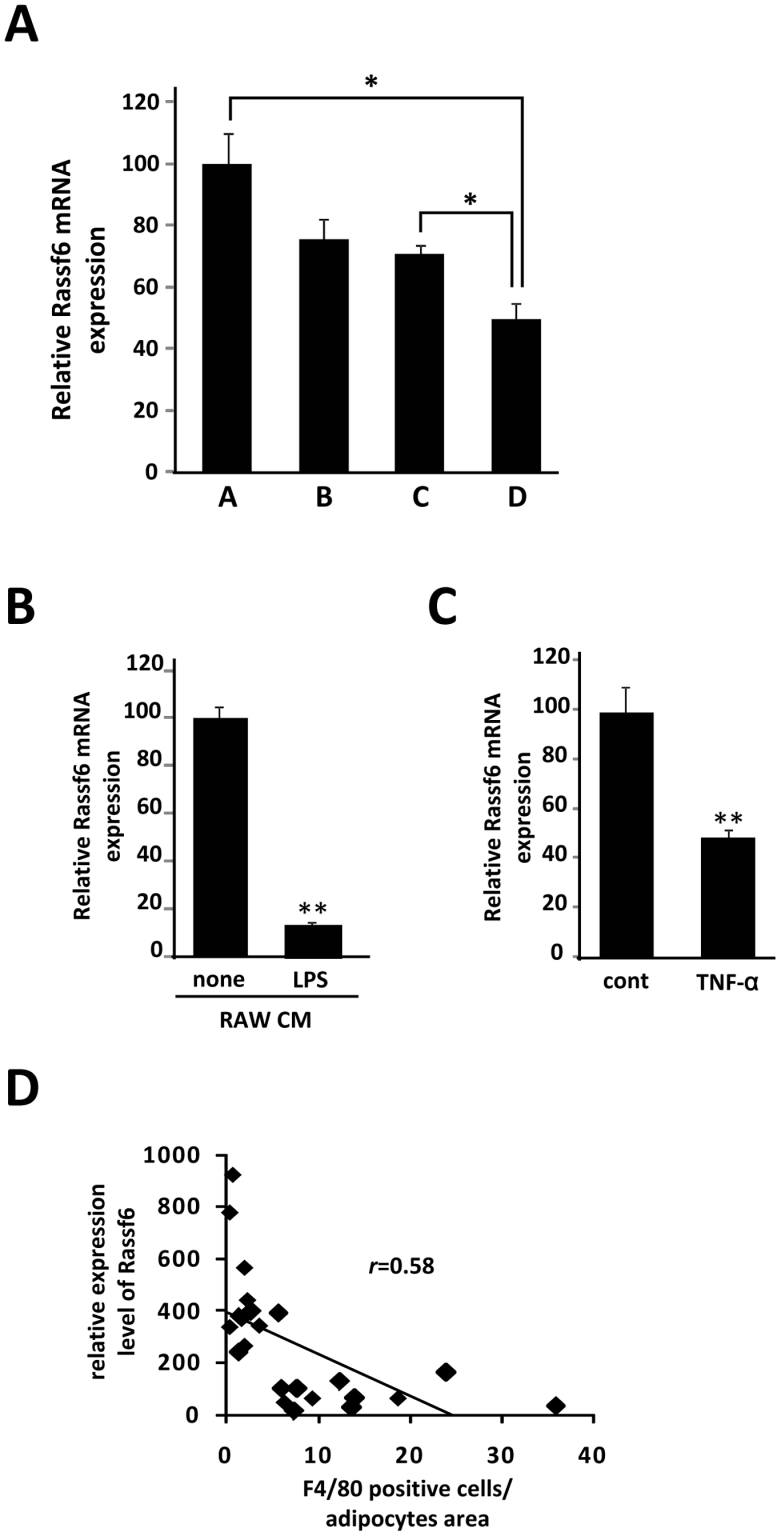
RASSF6 mRNA expression in adipocytes is affected in the presence of macrophages. *A*, Total RNAs from 4 groups (Fig. 4A) was extracted and subjected to quantitative PCR to examine mRNA expression level of *RASSF6*. All values are normalized to β-actin levels (n = 3). *P<0.05 compared with that of group A or C. *B*, Differentiated 3T3-L1 adipocytes were treated with conditioned medium of RAW264.7 cells without LPS (*none*) or stimulated in the presence of 1 µg/ml of LPS for 18 hr (*LPS*). The data (mean ± S.E.) are from a single experiment carried out (n = 3) and are representative of two independent experiments. *C*, Differentiated 3T3-L1 adipocytes were treated with 10 ng/ml of TNF-α for 24 h. Total RNAs were extracted and subjected to quantitative PCR analysis to examine expression level of RASSF6. The data (mean ± S.E.) are from a single experiment carried out (n = 3) and are representative of two independent experiments. **P<0.01. *D*, The relative mRNA expression level of RASSF6 gene was determined by quantitative PCR and normalized to β-actin level. Pearson's correlation coefficient showed a negative correlation between RASSF6 mRNA level and number of *F4/80* positive cells in adipose tissue of HFD mice.

We have shown an efficient strategy for the identification of adipocyte genes whose expression is increased as a consequence of interaction with infiltrated macrophages in adipose tissue *in vivo*. Conversely, of 1745 genes whose expression is significantly reduced in the db/db white adipose tissue (*p*<0.05, Fig. 4A), 18 genes were increased by dietary vitamin B6 (*p*<0.05, Table 3). Of these, we identified *RASSF6* (*Ras association domain family 6*) as a candidate gene. Whereas RASSF6 mRNA expression was markedly decreased in the adipose tissue of both db/db and HFD obese mice (Fig. 4B,C), it was up-regulated in response to vitamin B6 supplementation (Fig. 4D). As shown in Fig. 4E and 4F, RASSF6 mRNA was highly expressed in differentiated 3T3-L1 cells and mature adipocytes prepared from white adipose tissue of HFD obese mice. We further confirmed the down-regulation of RASSF6 mRNA level in 3T3-L1 adipocytes in the presence of RAW264.7 cells stimulated by LPS (Fig. 5A). Next, we examined the effect of conditioned medium of RAW264.7 cells on the RASSF6 mRNA expression in 3T3-L1 adipocytes, showing that conditioned medium of activated RAW264.7 cells can markedly reduce the RASSF6 mRNA in 3T3-L1 adipocytes (Fig. 5B). Moreover, as shown in Fig. 5C, TNFα was also able to suppress the RASSF6 expression, suggesting that activated RAW264.7 cells may suppress the RASSF6 mRNA expression in 3T3-L1 cells partially via TNFα-signaling. Most importantly, RASSF6 mRNA level and number of F4/80 positive cells in adipose tissue revealed a negative correlation (*r* = 0.769; *p*<0.01, Fig. 5D), suggesting a mechanistic link between RASSF6 mRNA expression in adipocytes and macrophage infiltration *in vivo*.

**Table 3:** Analysis of two transcriptomes to isolate genes whose expression is downregulated by the interaction with macrophages.

Gene name	Fold 1 (db/db/db/+)	Fold 2 (35 mg/1 mg B6)
Rassf6	0.10	2.17
AU018778	0.17	2.29
Il17rb	0.28	2.01
Fbxo21	0.23	2.03
Rarres1	0.42	2.52
9130218O11Rik	0.31	2.02

DNA microarray analysis was repeated with the Cy3 and Cy5 dyes reversed (a dye swap). Fold change (*Fold 1*) represents the average of mRNA expression level in db/db mice relative to db/+ mice. Fold change (*Fold 2*) represents the average of mRNA expression level in mice with a 35 mg PN HCl/kg diet relative to a 1 mg PN HCl/kg diet.

**Figure 6:**
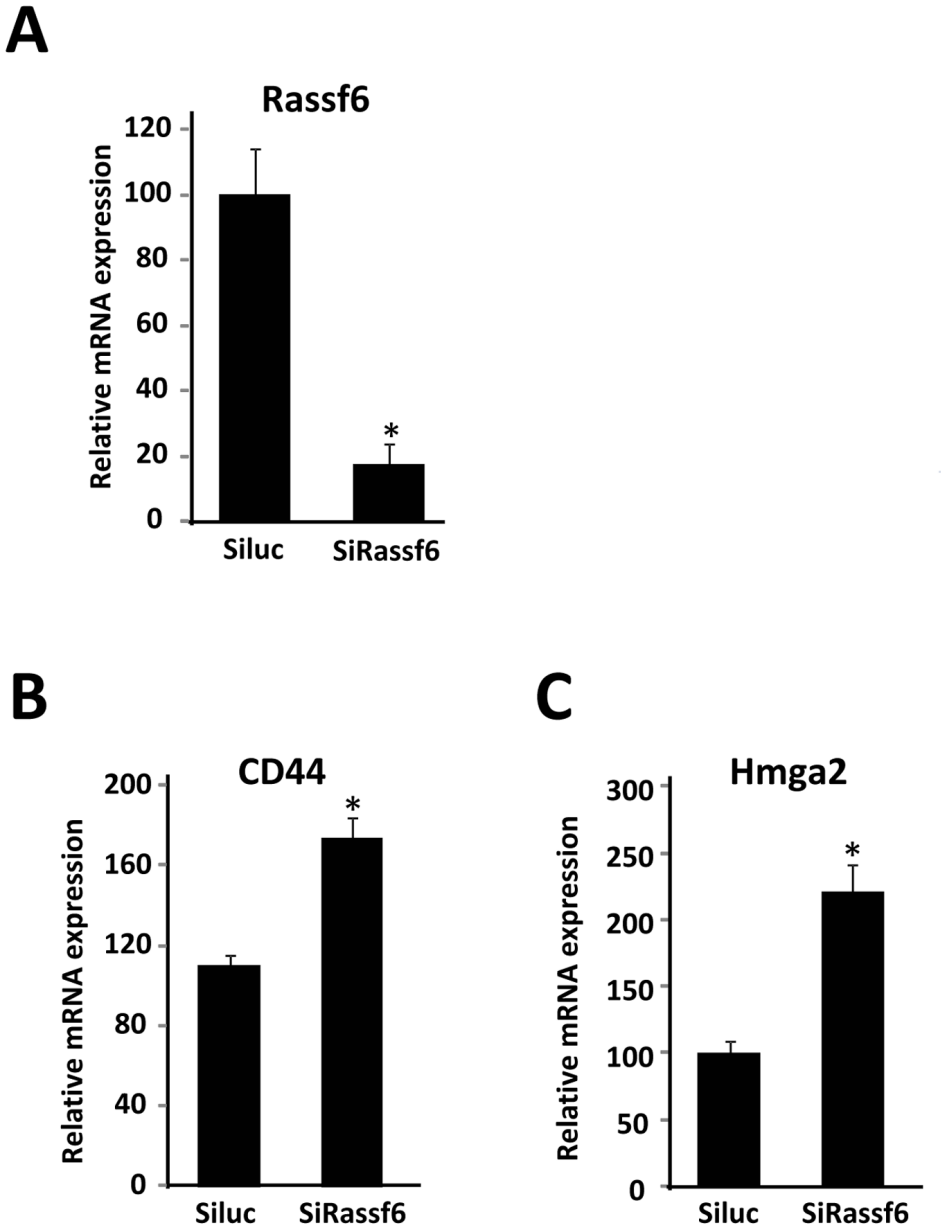
Effects of RASSF6 siRNA on gene expression in 3T3-L1 adipocytes. *A*–*C*, 3T3-L1 adipocytes were treated with MDI and differentiated into mature adipocytes as described under “Materials and Methods”. Differentiated 3T3-L1 adipocytes were transfected with luciferase siRNA (*Siluc*) or RASSF6 siRNA (*SiRassf6*). After 2 days of transfection, total RNAs were extracted and subjected to quantitative PCR analysis to examine expression levels of RASSF6, HMGA2 and CD44 mRNAs. The level of β-actin (*β-actin*) transcript was used as a control. *P<0.05 compared with those of cells transfected with control siRNA (*Siluc*).

To explore the biological significance of decreased RASSF6 expression in adipocytes, we used a small interference RNA-mediated RASSF6 knockdown strategy in 3T3-L1 adipocytes. The extent of the reduction in RASSF6 mRNA expression was assessed by quantitative RT-PCR (Fig. 6A). We performed DNA microarray analysis using RNA samples from control and RASSF6-deprived 3T3-L1 adipocytes. The reduction in RASSF6 mRNA expression did not affect the mRNA expression of adipogenesis-related genes, however, RASSF6 RNAi gene silencing resulted in enhanced expression of several genes in 3T3-L1 adipocytes (Table 4). As shown in Fig. 6B,C, quantitative RT-PCR assays confirmed a significant increase in CD44 and high mobility group protein HMGA2 gene expression in 3T3-L1 adipocytes.

**Table 4:** Effect of decreased RASSF6 expression on gene expression in 3T3-L1 adipocytes.

Gene ID	Gene symbol	Description	Fold	p Value
NM_009517	Zmat3	zinc finger matrin type 3	2.21	0.00
NM_198164	Cdc2l6	cell division cycle 2-like 6	2.15	0.00
NM_021443	Ccl8	chemokine (C-C motif) ligand 8	2.13	0.00
NM_010441	Hmga2	high mobility group AT-hook 2	2.10	0.00
NM_009196	Slc16a1	solute carrier family 16 (monocarboxylic acid transporters), member 1	2.02	0.00
NM_009851	Cd44	CD44 antigen	2.01	0.01
NM_026854	Dtwd2	DTW domain containing 2	1.88	0.00
NM_001003909	Ankib1	ankyrin repeat and IBR domain containing 1	1.85	0.00
NM_001081176	Polr3g	polymerase (RNA) III (DNA directed) polypeptide G	1.82	0.00
NM_023040	Gfer	growth factor, erv1 (S. cerevisiae)-like	1.81	0.00
NM_010068	Dnmt3b	DNA methyltransferase 3B	1.81	0.02
NM_025965	Ssr1	signal sequence receptor, alpha	1.79	0.00
NM_009741	Bcl2	B-cell leukemia/lymphoma 2	0.60	0.00
NM_010513	Igf1r	insulin-like growth factor I receptor	0.58	0.00
BC056501	Slc45a4	solute carrier family 45, member 4	0.56	0.00
NM_172416	Ostm1	osteopetrosis associated transmembrane protein 1	0.56	0.00
NM_029492	Zdhhc20	zinc finger, DHHC domain containing 20	0.54	0.00
XM_907304	Abcb7	ATP-binding cassette, sub-family B (MDR/TAP), member 7	0.53	0.00
NM_134114	Sft2d1	SFT2 domain containing 1	0.51	0.02
NM_027016	Tloc1	translocation protein 1	0.49	0.00
NM_008607	Mmp13	matrix metallopeptidase 13	0.43	0.00
NM_028478	Rassf6	Ras association (RalGDS/AF-6) domain family 6	0.23	0.00

DNA microarray analysis was repeated with the Cy3 and Cy5 dyes reversed (a dye swap), and fold change (*Fold*) represents the average of mRNA expression level in RASSF6-deprived 3T3-L1 adipocytes relative to control cells.

## Discussion

Although vitamin B6 is widely distributed in many foods, there is accumulating evidence that many adults are not obtaining adequate amounts of this vitamin from the diet, strongly suggesting that the nutritional significance of vitamin B6 is not adequately appreciated [26]. Vitamin B6 deficiency has been associated with inflammatory diseases, including rheumatoid arthritis (RA), asthma and cardiovascular diseases [19], [20], [27]. Previous studies have shown that low plasma vitamin B6 levels are associated with typical inflammatory chronic diseases, such as RA and inflammatory bowel diseases [28], [29]. In the current study, we showed that dietary vitamin B6 has the potential to improve a state of chronic, low grade inflammation in adipose tissue. In particular, dietary vitamin B6 suppressed macrophage infiltration into white adipose tissue without affecting the adipocyte size of white adipose tissue and systemic glucose tolerance, suggesting that the suppressive effect of dietary vitamin B6 on macrophage infiltration is carried out independently of adipocyte hypertrophy and insulin sensitivity. In this study, we showed that vitamin B6 supplementation down-regulates PTX3 and MMP3 expression in adipose tissue of mice. PTX3, a member of the pentraxin superfamily of acute phase proteins, is considered to be a novel immunoinflammatory marker that has been reported to associate with cardiometabolic risk factors and to predict adverse outcomes in individuals with cardiovascular disease [30]. The plasma MMP-3 level has been previously described to be a novel prognostic factor for future adverse cardiovascular events in stable coronary artery disease patients [31], [32]. Given that adequate dietary vitamin B6 intake has been demonstrated to have a protective role against cardiovascular disease [27], [33], our study raises the possibility that white adipose tissue is a primary target of dietary vitamin B6 to mediate protection against several diseases including arteriosclerosis.

In addition to lipid-laden, mature adipocytes, the SVF of adipose tissue is composed of various cell types including endothelial cells, adipose-derived stem cells/preadipocytes, fibroblasts, and immune cells. Among these cells, macrophage accumulation in adipose tissue is directly proportional to measures of adiposity in animals and humans [5], [8]. The percentage of F4/80 positive macrophages is significantly and positively correlated with both adipocyte size and body mass [8]. There is considerable evidence that CC motif chemokine receptor-2 (CCR2) and its major ligand MCP-1 (CCL2) play a role in macrophage infiltration into obese adipose tissue and that macrophages, when infiltrated, may participate in the inflammatory pathways that are activated in obese adipose tissue [9], [10]. The pathological role of interplay between adipocytes and macrophages in obese adipose tissue is of interest, because there is increasing evidence that infiltrated macrophages are involved in adipose tissue remodeling and chronic inflammation that may underlie the development of metabolic dysfunction and type 2 diabetes [1]–[3], [5]. *In vitro* coculture systems composed of adipocytes and macrophages were developed, and showed an interesting paracrine communication between these two types of cells. Suganami *et al.* showed that fatty acids released from hypertrophied adipocytes serve as ligand for Toll-like receptor (TLR) 4 on macrophages and potentially for an inflammatory and paracrine loop between these cell types [13]. Numerous pathological events during the course of obesity *in vivo* are considered to arise from the crosstalk between adipocytes and macrophages, but, at the molecular level, their potential roles *in vivo* remain unclear. Our aim in this study was to identify novel adipocyte-specific-genes that are differentially regulated by interaction with infiltrated macrophages using a stratified transcriptome gene enrichment approach. First, we performed a comparative analysis using gene expression profiling of obese db/db mice and their lean littermates, and we found numerous gene clusters differentially regulated during the course of obesity, consistent with previous studies [23], [24]. Secondly, of these genes, we identified candidate genes related to the increase in infiltrated macrophages in adipose tissue *in vivo* by comparing alterations of gene expression in response to vitamin B6 supplementation. Thirdly, we selected adipocyte-derived genes using our previous data [25] of gene expression profiles based on an *in vitro* coculture system using 3T3-L1 adipocytes and RAW264.7 macrophages. As we expected, PTX3, one of the identified genes, was positively correlated with the number of F4/80-positive macrophages in adipocytes and highly expressed in mature adipocyte fraction from adipose tissue. Although MMP3 expression in 3T3-L1 adipocytes was actually up-regulated in the presence of RAW264.7 cells, MMP3 mRNA was present in high amounts in the SV fraction of obese mice relative to WT mice. Therefore, it is possible that MMP3 may be still considered a macrophage marker such as CCL2 and Msr1.

Finally, we focused on the adipocyte-derived genes those are down-regulated in response to infiltrated macrophages in obese adipose tissue, because we expected that the crosstalk with infiltrated macrophages may cause some functional deficits in adipocytes *in vivo*. Our data showed that RASSF6, highly expressed in the mature adipocyte fraction from adipose tissue, was negatively correlated with the number of F4/80-positive macrophages. Interestingly, conditioned medium from activated RAW264.7 cells down-regulated RASSF6 mRNA expression in 3T3-L1 cells, and we speculate that the suppressive effect may be partially dependent on TNFα released from macrophages. RASSF6 was recently isolated as a member of the Ras-association domain family (RASSF), which comprises six members (RASSF1–6) with each harboring a RalGDS/AF-6 and Sav/RASSF/Hippo domain [34], [35]. Rassf6 is being increasingly recognized as an important tumor suppressor that is involved in cellular signaling pathways for cell apoptosis in various cell types including HeLa and MCF-7 cells [34], [35]. Ikebe et al. recently showed that RASSF6 has a remarkable function to antagonize Hippo signaling and to mediate apoptosis through a distinct pathway from the canonical Hippo pathway [36]. It is of great interest that RASSF family proteins are tumor suppressors that are frequently down-regulated during the development of human cancers [37]. A recent report also shows that RASSF6 is down-regulated in 30–60% of solid tumors [35]. Thus, evidence is accumulating that RASSF6 plays a role in tumourigenesis and likely functions as a regulator of apoptosis. On the other hand, RASSF reportedly regulates the TNFα signaling pathway. Sadoshima et al. showed that increased TNFα expression in hearts of mice lacking RASSF1A triggered cardiac fibroblast proliferation and promoted active fibrosis [38]. Rassf6 is also able to suppress NF-κB activation in A549 human lung tumor cells [35], suggesting that RASSF6 may be a critical regulator of adipose inflammation. As the physiological role of RASSF6 in adipocytes remains unclear, we explored the functional importance of down-regulation of RASSF6 expression in adipocytes by a small interference RNA in 3T3-L1 adipocytes. We showed that CD44 and high mobility group protein A2 (HMGA2) gene expression was up-regulated by the decrease in RASSF6 expression. CD44, an adhesion/homing molecule, is a major receptor for the glycosaminoglycan hyaluronan, which is one of the major components of the tumor extracellular matrix [39], [40], whereas HMGA2 is an architectural transcription factor that plays an important role in development and progression of malignant neoplasias [41]. HMGA2 is predominantly expressed in proliferating, undifferentiated mesenchymal cells and its expression is reportedly regulated by microRNA let-7 and BMP4 [42], [43]. Additionally, HMGA2 expression is related to a number of mesenchymal tumor cell types, including fat-cell tumors (lipomas) [44], [45]. CD44 and HMGA2 mRNA expression is actually increased in the adipose tissue of db/db obese mice as assessed by our DNA microarray analysis. Taken together, this suggests that the dramatic decrease in RASSF6 expression in obese adipose tissue could be involved in the control of the differentiation state and/or number of adipocytes during the course of obesity. This study provides new insights into the pathological roles of *in vivo* crosstalk between adipocytes and adipose macrophages. It also points to the potential biological importance of RASSF6 in adipocytes, not only during the onset of obesity, but also in other physiological processes such as white adipose tissue development and lipoma formation.

## Supporting Information

Figure S1 **Effect of high fat oil (20% corn oil) on mRNA expression of macrophage marker genes in adipose tissue.** 8 male CD-1 (ICR) mice (5 weeks old) were divided into 2 groups of 4 mice and fed the control diet (AIN-93G, *cont*) or a high fat diet (*HFD*). After 3 weeks, total RNAs were isolated from epididymal white adipose tissue using RNeasy lipid tissue kit (Qiagen Sciences, Germantown, MD) and subjected to quantitative PCR to examine mRNA expression level of selected genes.
(DOCX)

Figure S2 **Dietary vitamin B6 did not affect the adipocyte size of epididymal white adipose tissue.** Epididymal white adipose tissue from mice (n = 6) were fixed in 10% buffered formalin and imbedded in paraffin. Multiple sections (separated by 70–80 µm each) were obtained and analyzed systematically with respect to adipocyte size. Staining of the sections was performed with hematoxylin and eosin. At least 5 fields (representing ∼100 adipocytes)/mice were analyzed.
(DOCX)

Figure S3 **Effect of vitamin B6 supplementation on glucose tolerance in mice.** At the end of feeding period, mice (n = 5) were feed deprived for 8 h and glucose tolerance tests were performed. 2 g/kg body weight of 20% D-glucose was injected intraperitoneally. Tail blood was taken at 0, 15, 30, 60, and 120 min after the injection, and used to measure the blood glucose levels using with a commercially available kit (Glucose test Wako, Wako Pure Chemical Industries, Japan).
(DOCX)

Table S1 **Effect of dietary level of vitamin B6 (pyridoxine HCl) on tissue concentration of pyridoxal 5′-phosphate (PLP) in mice fed 20% corn oil diet.** Values represent means ± S.E. (n = 4). **p<0.05 compared with that of 1 mg/kg vitamin B6.
(DOCX)

Table S2 **Body weight, food intake and adipose tissue weight.** Values represent means ± S.E. (n = 12).
(DOCX)
