# Supplementary material for: RASSF6 Expression in Adipocytes Is Down-Regulated by Interaction with Macrophages
Source: PLoS One. 2013 Apr 23;8(4):e61931. doi: 10.1371/journal.pone.0061931 (PMC3633988; doi:10.1371/journal.pone.0061931)
Supplement: Supplementary file 1 [file pone.0061931.s001.docx]

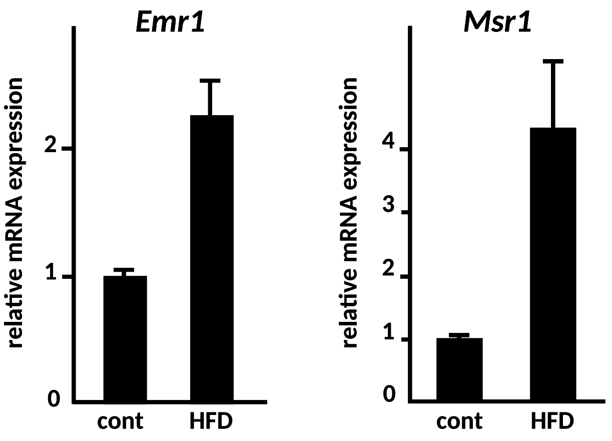
Emr1
relative mRNA expression
0
1
2
cont
HFD
Msr1
relative mRNA expression
0
1
2
3
4
cont
HFD
