# Supplementary material for: RASSF6 Expression in Adipocytes Is Down-Regulated by Interaction with Macrophages
Source: PLoS One. 2013 Apr 23;8(4):e61931. doi: 10.1371/journal.pone.0061931 (PMC3633988; doi:10.1371/journal.pone.0061931)
Supplement: Supplementary file 2 [file pone.0061931.s002.docx]

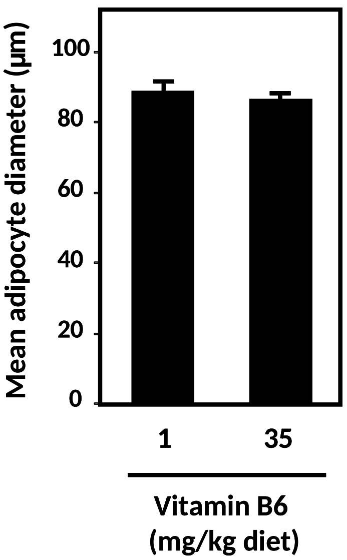

Mean adipocyte diameter (µm)
100
80
60
40
20
0
1
35
Vitamin B6
(mg/kg diet)
