# Supplementary material for: RASSF6 Expression in Adipocytes Is Down-Regulated by Interaction with Macrophages
Source: PLoS One. 2013 Apr 23;8(4):e61931. doi: 10.1371/journal.pone.0061931 (PMC3633988; doi:10.1371/journal.pone.0061931)
Supplement: Supplementary file 3 [file pone.0061931.s003.docx]

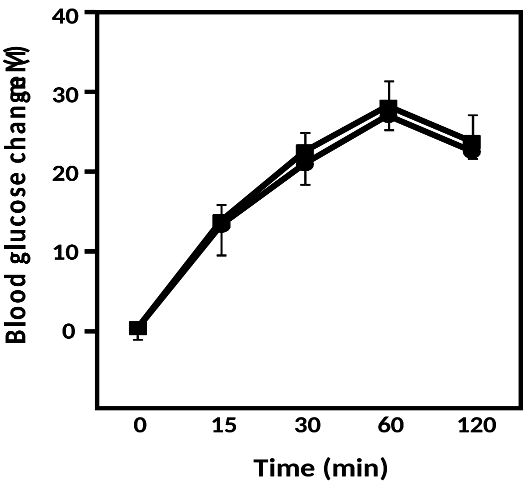

40
30
20
10
0
Blood glucose change (mM)
0
15
30
60
120
Time (min)
