# Supplementary material for: RASSF6 Expression in Adipocytes Is Down-Regulated by Interaction with Macrophages
Source: PLoS One. 2013 Apr 23;8(4):e61931. doi: 10.1371/journal.pone.0061931 (PMC3633988; doi:10.1371/journal.pone.0061931)
Supplement: Supplementary file 4 [file pone.0061931.s004.docx]

	vitamin B6 (mg/kg diet)			
	0	1	7	35
nmol/ g adipose tissue	0.54 ± 0.06	0.60 ± 0.04	1.27 ± 0.10	2.54 ± 0.48**
