# Supplementary material for: RASSF6 Expression in Adipocytes Is Down-Regulated by Interaction with Macrophages
Source: PLoS One. 2013 Apr 23;8(4):e61931. doi: 10.1371/journal.pone.0061931 (PMC3633988; doi:10.1371/journal.pone.0061931)
Supplement: Supplementary file 5 [file pone.0061931.s005.docx]

	vitamin B6 (mg/kg diet)	
	1	35
BW, *g*	50.9 ± 0.9	48.6 ± 0.9
Food intake, *g/d*	3.87 ± 0.05	4.04 ± 0.04
Adipose tissue weight, *g*	2.36 ± 0.12	2.35 ± 0.11
